# Heading perception from optic flow is affected by heading distribution

**DOI:** 10.1177/20416695221133406

**Published:** 2022-11-22

**Authors:** Qi Sun, Ruifang Yan, Jingyi Wang, Xinyu Li

**Affiliations:** Department of Psychology, 66344 Zhejiang Normal University, Jinhua, People’s Republic of China; Key Laboratory of Intelligent Education Technology and Application of Zhejiang Province, Zhejiang Normal University, Jinhua, People’s Republic of China; Department of Psychology, 66344 Zhejiang Normal University, Jinhua, People’s Republic of China; Department of Psychology, 66344 Zhejiang Normal University, Jinhua, People’s Republic of China; Department of Psychology, 66344 Zhejiang Normal University, Jinhua, People’s Republic of China; Key Laboratory of Intelligent Education Technology and Application of Zhejiang Province, Zhejiang Normal University, Jinhua, People’s Republic of China

**Keywords:** heading perception, optic flow, central tendency, heading distribution effect

## Abstract

Recent studies have revealed a central tendency in the perception of physical features. That is, the perceived feature was biased toward the mean of recently experienced features (i.e., previous feature distribution). However, no study explored whether the central tendency was in heading perception or not. In this study, we conducted three experiments to answer this question. The results showed that the perceived heading was not biased toward the mean of the previous heading distribution, suggesting that the central tendency was not in heading perception. However, the perceived headings were overall biased toward the left side, where headings rarely appeared in the right-heavied distribution (Experiment 3), suggesting that heading perception from optic flow was affected by previously seen headings. It indicated that the participants learned the heading distributions and used them to adjust their heading perception. Our study revealed that heading perception from optic flow was not purely perceptual and that postperceptual stages (e.g., attention and working memory) might be involved in the heading perception from optic flow.

Gibson (1950) proposed that observers could rely on the optic flow—a dynamic light motion pattern projected on observers’ retina as observers move in an environment—to judge their self-motion direction (i.e., heading), which was supported by many studies (Gibson 1950; Longuet-Higgins & Prazdny 1980; Nakayama & Loomis 1974; Royden & Moore, 2012; Warren & Rushton, 2009). Several studies revealed that when the optic flow simulated observers moving straight forward, observers could accurately perceive their heading directions within 1°–2° errors by localizing the center of the flow field known as the focus of expansion (FoE) (Crowell & Banks, 1996; Li et al., 2002; Warren, 1976; Warren et al., 1988, 2001).

Although heading perception from an optic flow can be very precise, the perceived heading (PH) from the optic flow is systematically compressed to the ego-centric direction (i.e., head–body direction) or the straight-ahead direction that we most frequently move along, known as center bias (Li et al., 2002; Sun & Li, 2019; Sun et al., 2020; Warren & Hannon, 1988; Warren & Kurtz, 1992; Warren & Saunders, 1995; Xing & Saunders, 2016). For example, Sun and Li (2019) found that as the observer's ego-centric direction was right or left of the display center, the PH was biased toward the ego-centric direction. Recent neurophysiological work has also revealed that the neurons of area medial superior temporal area (MST) and ventral intra-parietal area (VIP) that respond to the shift of heading direction simulated by optic flow take the ego-centric direction as the reference to encode the heading (Chen et al., 2011, 2018). Specifically, when monkeys’ ego-centric direction was shifted, the activities of some neurons in these areas could be changed. Additionally, previous studies generally propose that the center bias in the heading direction is consistent with a Bayesian inference account in which the ego-centric direction works as a prior. When the reliability of optic flow decreases, observers will rely more on the ego-centric direction, showing a stronger center bias (Sun & Li, 2019; Sun et al., 2020; Xing & Saunders, 2016).

In addition to center bias, recent work has shown another perception error, central tendency. Central tendency means that the current feature estimate is systematically compressed to the mean of the previously seen features (i.e., previous feature distribution) (Hollingworth, 1910). As the mean of the previous distribution was shifted, the feature estimate would be systematically compressed to the distribution mean. For example, Jazayeri and Shadlen (2010) asked participants to finish three sessions of the time interval reproducing task. In each session, time intervals were selected from one of three partially overlapping discrete uniform previous distributions (i.e., “Short,” “Intermediate,” and “Long” as shown by the black, dark red, and light red bar charts in Figure 1a). The means of the three previous distributions were different. The results showed that the production time (i.e., perceived time intervals) was systematically compressed toward the mean of the previous distribution (Figure 1b) (also see Ryan, 2011). A similar central tendency pattern is also observed in the perception of line length (Ashourian & Loewenstein, 2011; Duffy et al., 2010; Huttenlocher et al., 2000), facial expressions (Roberson et al., 2007; Corbin et al., 2017), absolute size (Huttenlocher et al., 2000), and color (Olkkonen & Allred, 2014; Olkkonen et al., 2014). Researchers generally proposed that the central tendency reflects the ability of the central neural system to temporally integrate recent experiences, and the postperceptual stage (e.g., working memory) is involved in the feature perception. Additionally, several studies found that as the reliability of physical features decreased, perceived features were more biased toward the mean of the previous distribution, showing a stronger central tendency. Hence, researchers proposed that central tendency was consistent with a Bayesian inference account in which the mean of the previous distribution worked as the prior (Ashourian & Loewenstein, 2011; Jazayeri & Shadlen, 2010; Olkkonen & Allred, 2014; Olkkonen et al., 2014).

**Figure 1.**
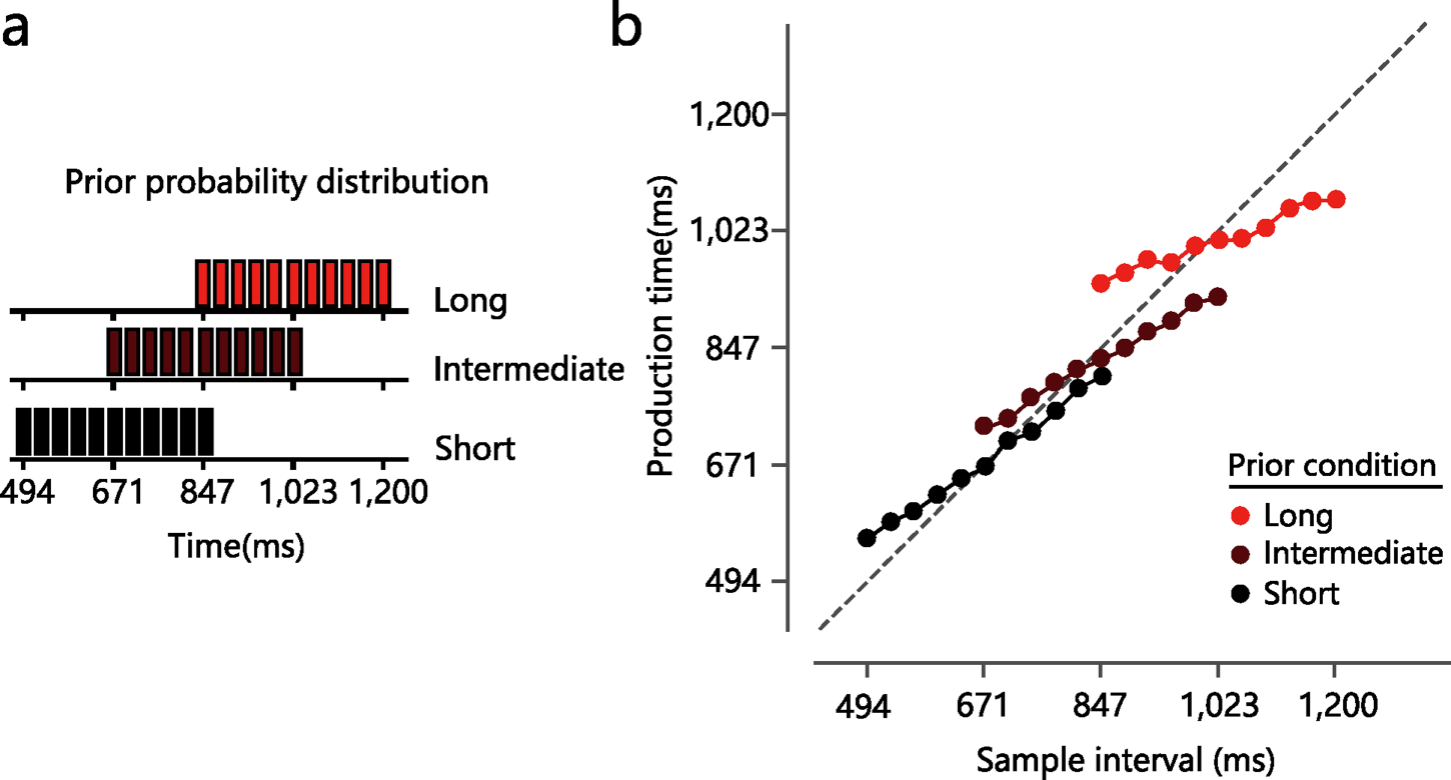
Prior (or previous) distributions and the main results of Jazayeri and Shadlen (2010). (a) Three prior (or previous) distributions of time intervals. (b) Production time (i.e., perceived time interval) against the sample time interval. The dashed diagonal denotes the ideal performance meaning that the production time is equal to the sample time. The horizontal dash line indicates the pure central tendency meaning that the production time is always the mean of the previous distribution regardless of the sample interval. The graph shows that the production time is systematically biased toward the mean of the previous distribution. The two are edited based on Figure 1b and Figure 2 in Jazayeri and Shadlen (2010).

Xing and Saunders (2016) first examined the effects of previous heading distributions on the heading perception from optic flow. They showed participants two previous heading distributions: uniform versus M-shape distribution. In the M-shape distribution, the number of trials increased with the heading. It was found that the main effect of heading distribution was not significant. Accordingly, they reported that participants did not learn the previous heading distributions. They, therefore, concluded that heading perception from optic flow was purely perceptual. However, the means of the two previous heading distributions were both 0° aligned with the ego-centric direction, which made it unclear whether a central tendency was in heading perception. If there was a central tendency, then observers could learn the previous heading distributions and use it to adjust their performance, suggesting that heading perception from optic flow was not purely perceptual.

In the current study, we conducted three experiments to examine whether and how the heading distribution that participants previously learned (i.e., previous heading distribution) affected heading perception from optic flow. If previous heading distribution affected heading perception, then PHs would be systematically compressed to the distribution mean, showing a central tendency. In Experiments 1 and 2, we presented participants with uniform distributions with different distribution widths and means. If PHs were significantly different between different previous uniform distributions, then previous heading distributions affected heading perception from optic flow, suggesting that participants learned the previous heading distribution and adopted the previous heading distribution to adjust their heading performances. Especially, if PHs were systematically compressed to the mean of previous heading distributions, then a central tendency was in heading perception. Our results of Experiments 1 and 2 showed that PHs were not significantly different between different uniform distributions, indicating that heading perception was not affected by the uniform distribution.

Previous studies proposed that central tendency was consistent with the Bayesian inference account in which the previous feature distributions work as the priors (Ashourian & Loewenstein, 2011; Jazayeri & Shadlen, 2010; Olkkonen & Allred, 2014; Karaminis et al., 2016). Theoretically, this account could explain the finding of Experiment 1. In this model, we introduced a prior to capture the previous/learned information about (1) ego-centric direction and (2) heading distribution. The posterior distribution can be given by:

(1) $$p(X|M_{i})=\frac{\;p(M_{i}|X)p(X|\theta )}{\sum_{i}^{n}\;p(M_{i}|X)p(X|\theta )}$$

where $p(M_{i}|X)$ indicated the likelihood, $M_{i}$ was the PH of *i*^th^ trial as the actual heading (AH) was *X*; p(X|θ) indicated the prior and was the product of the distribution of ego-centric direction and previous headings. That is, $p(X|\theta )=p_{\mathrm{ego}}(X|\theta_{\mathrm{ego}})\times p_{\mathrm{dis}}(X|\theta_{\mathrm{dis}})$. $p_{\mathrm{ego}}(X|\theta_{\mathrm{ego}})$ indicated the prior of the ego-centric direction, and $\theta_{\mathrm{ego}}$ indicated the ego-centric direction; $p(X|\theta_{\mathrm{dis}})$ indicated the prior of the heading distribution, and $\theta_{\mathrm{dis}}$ indicated the center of the previous heading distribution. Therefore, equation (1) can be expressed as:

(2) $$p(X|M_{i})=\frac{\;p(M_{i}|X)p_{\mathrm{ego}}(X|\theta_{\mathrm{ego}})p_{\mathrm{dis}}(X|\theta_{\mathrm{dis}})}{\sum_{i}^{n}\;p(M_{i}|X)p_{\mathrm{ego}}(X|\theta_{\mathrm{ego}})p_{\mathrm{dis}}(X|\theta_{\mathrm{dis}})}$$

When the previous heading distribution was uniform, participants might encode that the probabilities of all headings were the same (e.g., $p_{\mathrm{dis}}(X|\theta_{\mathrm{dis}})=1/66$). Hence, equation (2) can be converted into:

(3) $$p(X|M_{i})=\frac{\;p(M_{i}|X)p_{\mathrm{ego}}(X|\theta_{\mathrm{ego}})}{\sum_{i}^{n}\;p(M_{i}|X)p_{\mathrm{ego}}(X|\theta_{\mathrm{ego}})}$$

Therefore, equation (3) showed that, given a uniform heading distribution, participants’ PHs were only affected by the ego-centric direction. To address this question, we showed participants uniform and right-heavied nonuniform heading distributions in Experiment 3, in which there were more trials for headings that were right to the ego-centric direction than headings that were left to the ego-centric direction. As a result, the distribution probabilities ($p_{\mathrm{dis}}(X|\theta_{\mathrm{dis}})$) were varied among headings, and $p_{\mathrm{dis}}(X|\theta_{\mathrm{dis}})$ could not be removed from equation (2). Therefore, we expected that nonuniform distributions could affect heading perception from optic flow. Our results showed that PHs were significantly different between the uniform and right-heavied distributions, but what differed from central tendency was that PHs were overall biased away from the mean of the right-heavied distribution. The finding suggested that heading perception from optic flow could be affected by previous heading distribution, indicating that participants learned the previous heading distribution and postperceptual stages (e.g., working memory) were involved in heading perception from optic flow. Our study first revealed the involvement of postperceptual stages in heading perception, which was inconsistent with the idea of Xing and Saunders (2016) that heading perception from optic flow was purely perceptual.

## Experiment 1. Uniform Distribution Does not Affect Heading Perception From Optic Flow

**Figure 2.**
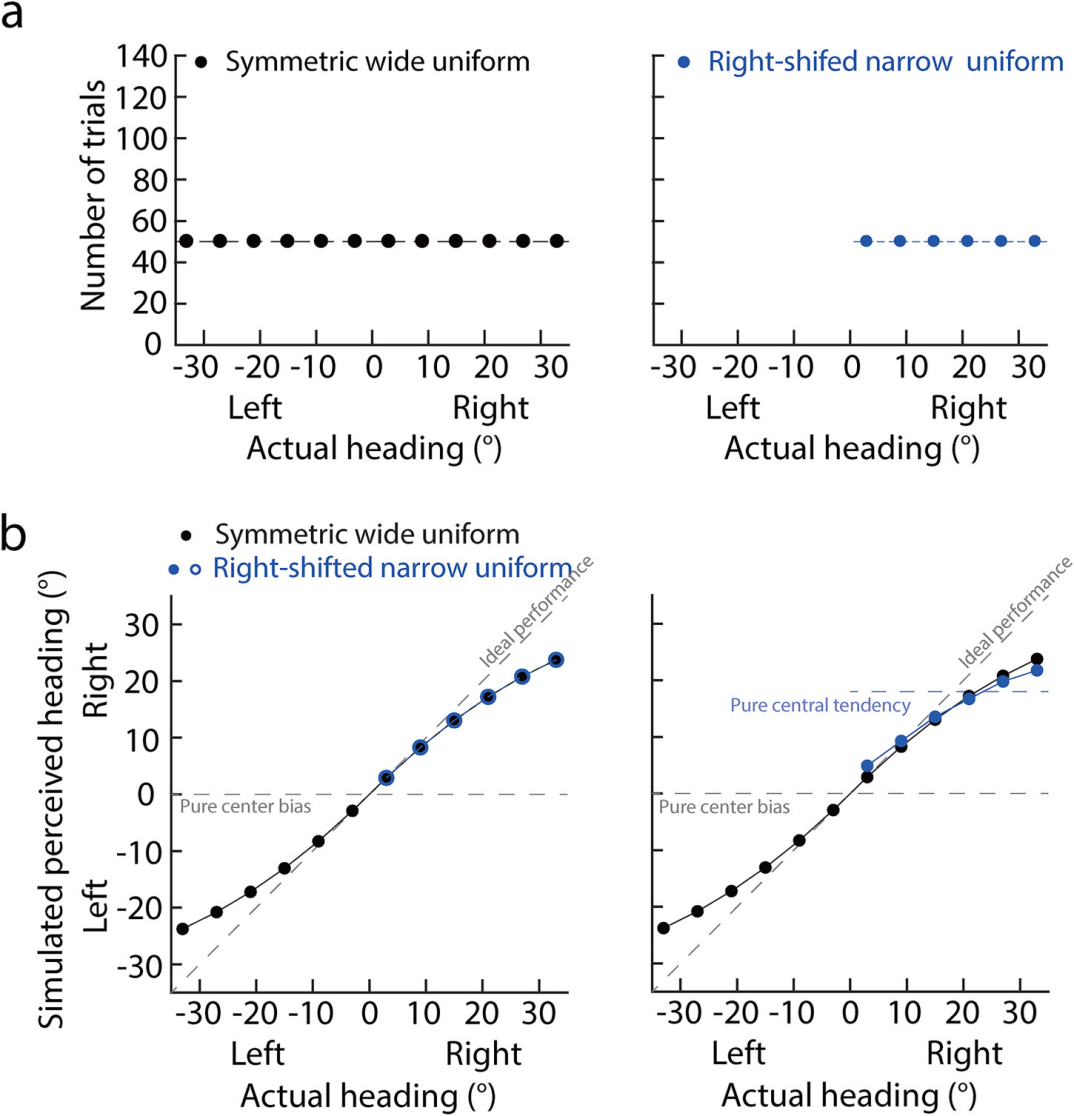
(a) Number of trials against the actual heading. Left and right panels show symmetric wide and right-shifted narrow uniform distributions. (b) Simulated perceived heading against actual heading. The diagonal dashed gray line illustrates the ideal performance, meaning that observers can accurately perceive headings. The horizontal dashed gray line illustrates the pure center bias performance, meaning that observers consistently report 0° (i.e., ego-centric direction) regardless of actual headings. The blue dashed line in the right panel illustrates the pure central tendency in the right-shifted narrow uniform distribution, meaning that observers consistently reported the distribution mean regardless of actual headings. In the symmetric wide uniform distribution, simulated perceived headings are compressed to the ego-centric direction (black dots). The blue circles in the left panel show simulated perceived headings in the right-shifted narrow heading distribution when the heading distribution did not affect heading perception. The blue dots in the right panel show simulated perceived headings in the right-shifted narrow heading distribution when the central tendency was in heading perception.

Experiment 1 examined whether and how previous heading distributions affected heading perception from optic flow. Participants completed heading perception tasks in two uniform distribution conditions: symmetric wide and right-shifted narrow uniform distributions. The headings in the symmetric wide uniform distribution (left panel in Figure 2a) were selected from a wide range and symmetric about the ego-centric direction (0°). All headings in the right-shifted narrow uniform distribution (right panel in Figure 2a) were selected from a narrow range and all right to the ego-centric direction (0°). Therefore, the means of the two distributions were separated. Based on the findings of previous center bias studies, PHs in the symmetric wide uniform distribution would be systematically compressed to the ego-centric direction (0°) (black dots in the left panel of Figure 2b). If the previous heading distribution did not affect heading perception and only ego-centric direction affected heading perception, then the PHs in the two distributions would be superimposed (blue circles and black dots in the left panel of Figure 2b). In contrast, if both center bias and central tendency were in heading perception, the distribution mean would have an extra attractive effect on PHs. Due to the separation of the ego-centric direction and distribution mean in the right-shifted heading distribution, the headings that were far away from the distribution mean would show a bias toward the distribution mean (dashed blue line). As a result, the PHs in the symmetric wide distribution would be separated from that in the right-shifted narrow distribution (blue and black dots in the right panel of Figure 2b).

### Methods

#### Participants

Twenty participants (12 females, 8 males, aged 19–23 years) were recruited from Zhejiang Normal University. All were naïve to the experimental purpose and had a normal or corrected-to-normal vision. The experiment was approved by the Scientific and Ethical Review Committee in the Department of Psychology of Zhejiang Normal University.

#### Stimuli and Apparatus

**Figure 3.**
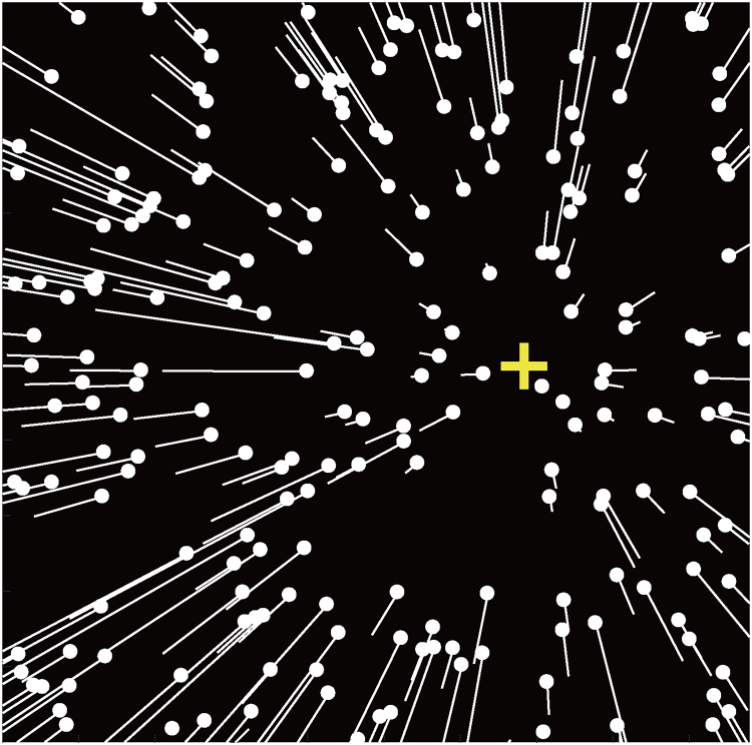
Schematic illustration of visual stimuli used in the current study. White dots illustrate the dots’ initial positions at the first frame of each display; the white lines illustrate the dots’ motion trajectories in the following frame, which are invisible in the experiment. Yellow “+” indicates the heading direction and is not shown in the experimental stimuli.

The display (Figure 3) simulated observers translating at 1.5 m/s through a 3D dot cloud (80° H × 80° V, depth range: 0.20–5 m; eye height: 0.17 m) consisting of 126 dots (diameter: 0.28°). The simulated self-motion direction (i.e., heading direction) of each display was randomly selected from ±33°, ±27°, ±21°, ±15°, ±9°, or ±3°. Positive and negative values corresponded to heading to the right or left of the ego-centric direction (i.e., 0°), respectively.

The displays were programmed in MATLAB using the Psychophysics Toolbox 3 and presented on a 27-inch Dell monitor (resolution: 2560 H × 1440 V pixels; refresh rate: 60 Hz) with an NVIDIA GeForce GTX 1660Ti graphics card.

#### Procedure

All participants sat in a light-excluded room with their heads stabilized with a chin-rest at a viewing distance of 20 cm. They viewed displays monocularly with their right eye to reduce the conflict between motion parallax (indicating a 3D moving stimulus) and binocular disparity (indicating a flat 2D display screen) depth cues. Before the start of the experiment, participants’ ego-centric direction was aligned with the display center. Before the start of each block, one fixation point was presented at the center of the display, and participants were asked to fixate on the fixation point and maintain their eye position there throughout the experiment. Then they could press the space key to start the experiment.

On each trial, the simulated self-motion display was presented for 500 ms followed by a blank display with a horizontal line that appeared across the mid-section of the display. Participants were asked to move a mouse-controlled probe to indicate their PH along the horizontal line. When the participants clicked the mouse button, the next trial started immediately.

Participants performed two blocks of trials. Each block corresponded to one heading distribution: symmetric wide or right-shifted narrow uniform conditions (Figure 2a). In the symmetric wide uniform distribution, headings were randomly selected from ±33°, ±27°, ±21°, ±15°, ±9°, or ±3°, so the mean of the distribution was 0° aligned with the ego-centric direction (0°). In the right-shifted narrow uniform condition, headings were randomly selected from 3°, 9°, 15°, 21°, 27°, or 33°. All headings were right to the ego-centric direction, and the mean of the distribution was 18° right to the ego-centric (0°). Each heading was repeated for 50 trials leading to 600 and 300 trials in the two blocks, respectively.

Before the start of the experiment, participants were given 20 practice trials to familiarize themselves with the experiment. The headings of the practice trials were randomly selected from ±33°, ±27°, ±21°, ±15°, ±9°, or ±3°. The conducting sequences of the two blocks were counterbalanced among participants. Participants took about 50 min to finish the experiment.

### Results and Discussion

We first examined whether center bias was in heading perception by fitting the PH as a linear function of the AH (Sun et al., 2020). If the slope was <1, then headings were systematically underestimated and compressed to the ego-centric direction, showing a center bias in heading perception. One sample *t*-test showed that the slopes of the symmetric wide and right-shifted narrow distributions were all significantly smaller than 1 (mean ± SE: 0.79 ± 0.031, *t*[19] = −6.79, *p* < .001, Cohen's *d* = 1.52; 0.83 ± 0.043, *t*[19] = −3.96, *p* < .001, Cohen's *d* = 0.89). These slopes were close to those of Sun et al. (2020), in which they found the slope was around 0.71 (Experiment 1), and Xing and Saunders (2016), in which the slope was around 0.8.

**Figure 4.**
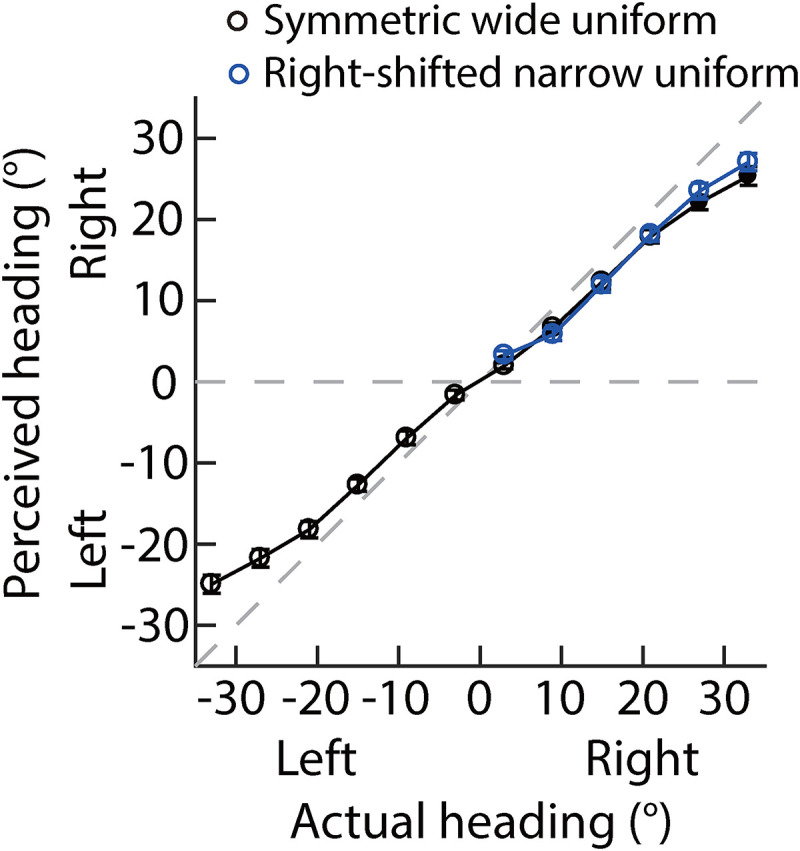
Results of experiment 1. The perceived heading (*y*-axis) was against the actual heading (*x*-axis). The left and right on the *x*-axis (*y*-axis) mean the actual heading (perceived headings) was left or right in the ego-centric direction (0°). Each circle represents the mean perceived heading averaged across 20 participants; error bars represent the standard error across 20 participants.

Figure 4 plots the PH against the AH. It clearly shows that when the AHs are right in the ego-centric direction, the PHs in the right-shifted narrow uniform distribution (blue circles) and the symmetric wide uniform distribution (black circles) are nearly perfectly superimposed. A 2 distributions (symmetric wide uniform vs. right-shifted narrow uniform) × 6 headings (from 3° to 33° by steps of 6°) repeated measures analysis of variance (ANOVA) showed that the main effects of distributions were not significant (*F*[1,19] = 1.13, *p* = .30, *η*^2^ = 0.056), but its interaction with headings was significant (Greenhouse–Gessier corrected: *F*[2.99, 57.76] = 3.27, *p* = .028, *η*^2^ = 0.16). Further post hoc analysis showed that the PHs were not significant between two uniform distributions for most of the AHs (*p*s > .057), except for the AH of 3° (*p* = .045).

To summarize, Experiment 1 showed that regardless of the previous heading distributions, a center bias was in heading perception from optic flow, consistent with previous studies (Sun et al., 2020; Xing & Saunders, 2016). Additionally, the previous uniform distributions did not affect the heading perception from optic flow. Specifically, the PH was not biased toward the mean of the previous heading distribution, suggesting that the central tendency was not in the heading perception from optic flow. This finding implied that the heading perception from optic flow differed from other physical feature perceptions involving the central tendency effect (Ashourian & Loewenstein, 2011; Corbin et al., 2017; Olkkonen & Allred, 2014; Olkkonen et al., 2014).

## Experiment 2. Reexamination of Experiment 1

**Figure 5.**
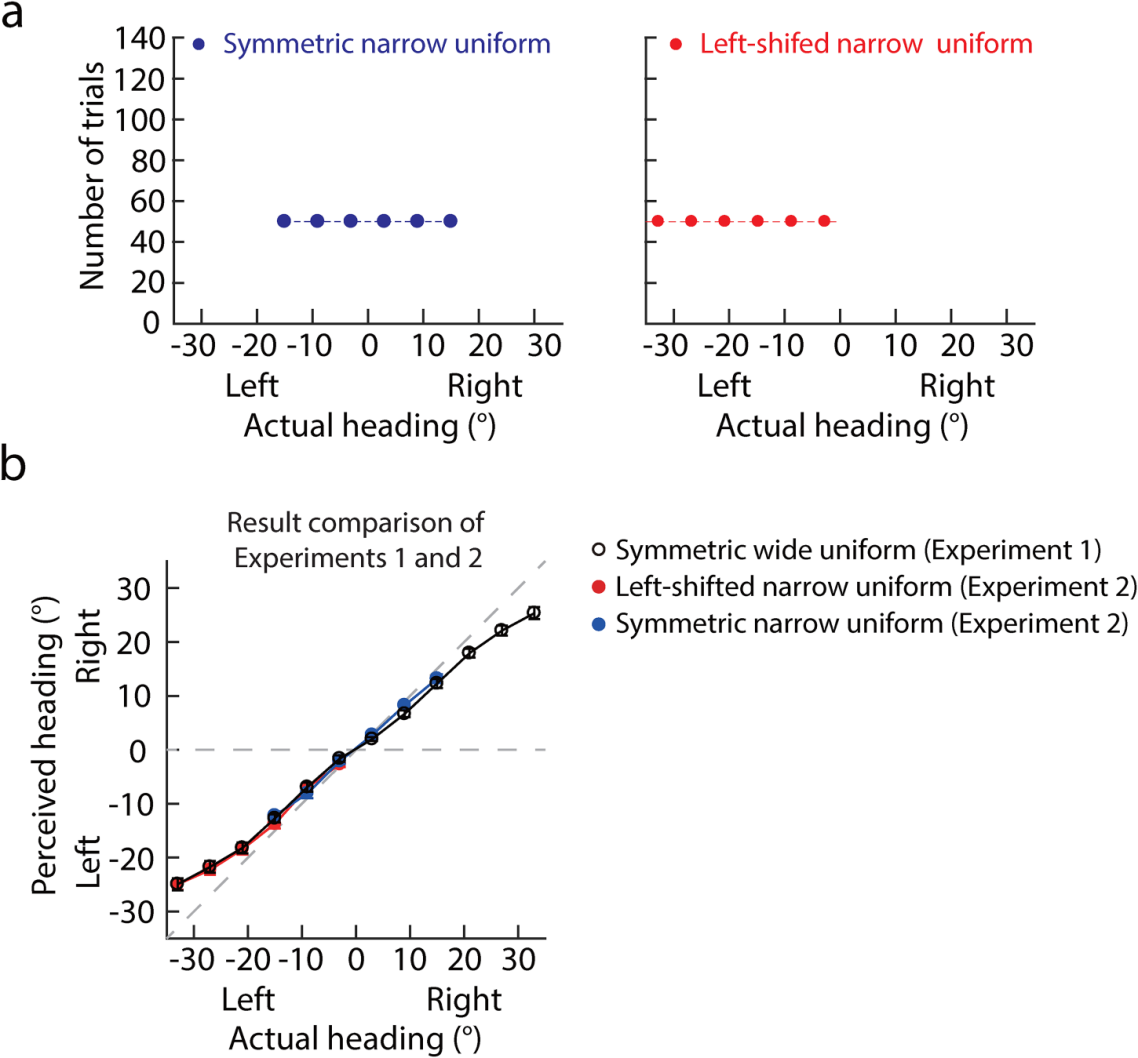
(a) Number of trials against the actual heading. The left and right panels show symmetric wide and right-shifted narrow uniform distributions. (b) Result comparison of Experiments 1 and 2. The perceived heading (*y*-axis) was against the actual heading (*x*-axis). The left and right on the *x*-axis (*y*-axis) mean the actual heading (perceived headings) was left or right in the ego-centric direction (0°). Each circle represents the mean perceived heading averaged across 20 participants; error bars represent the standard error across 20 participants. Black circles and red and blue dots indicate the mean perceived headings in the symmetric wide uniform distribution (Experiment 1), left-shifted and symmetric narrow uniform distributions (Experiment 2).

Experiment 1 found that the previous uniform heading distribution did not affect heading perception from optic flow. However, the two distributions were with different widths (one was [−33°, 33°]; the other was [3°, 33°]). Compared with the narrow distribution, the wide distribution increased the uncertainty of headings. According to Bayesian inference theory, high uncertainty of headings would increase the center bias size (e.g., Sun et al., 2020), which might reduce the separation of the PHs in the two distributions of Experiment 1. Therefore, no effect of heading distribution on heading perception might be due to the different distribution widths. To address this question, Experiment 2 showed 20 new participants (14 females, 6 males, aged 18–24 years) two narrow uniform distributions: symmetric and left-shifted narrow uniform distributions (Figure 5a). The headings of the symmetric narrow uniform distribution were randomly selected from a narrow range of [−15°, 15°] and symmetric about the ego-centric direction (0°). The headings of the right-shifted narrow uniform distribution were selected from a narrow range of [−33°, −3°] and all left to the ego-centric direction (0°). The stimulus parameters and experimental methods were all similar to that of Experiment 1. Participants took about 30 min to finish this experiment.

### Results and Discussion

Like Experiment 1, we first fitted the PH as a linear function of the AH to examine whether center bias was in heading perception. One sample *t*-test showed that the slopes of the symmetric and left-shifted narrow uniform distributions were all significantly smaller than 1 (mean ± SE: 0.86 ± 0.050, *t*[19] = −2.79, *p* = .012, Cohen's *d* = 0.62; 0.77 ± 0.034, *t*[19] = −6.82, *p* < .001, Cohen's *d* = 1.52), indicating a center bias in heading perception, consistent with Experiment 1.

Figure 5b shows the comparisons of PHs between Experiments 1 and 2. The black circles show the PHs of the symmetric wide uniform distribution in Experiment 1. The red and blue circles show the PHs of the left-shifted and symmetric narrow uniform distributions in Experiment 2. The circles of the three distributions are nearly perfectly superimposed. We examined the differences in PHs between symmetric wide and left-shifted narrow, symmetric wide and narrow distributions, respectively, with one 2 distributions (Experiments 1 and 2) × 6 headings ([−33°, −3°] or [−15°, 15°]) mixed repeated measures ANOVA. The results showed that neither the main effects of distributions nor its interaction with headings was significant as the headings were left to the straight-ahead direction (*F*[1,38] = 0.77, *p* = .68, *η*^2^ = 0.0046; *F*[5,190] = 0.50, *p* = .78, *η*^2^ = 0.0065), and in a narrow range (*F*[1,38] = 0.19, *p* = .66, *η*^2^ = 0.0050; *F*[5,190] = 1.17, *p* = .33, *η*^2^ = 0.033).

To sum up, the current experiment with different participants and narrow distributions well reproduced the findings of Experiment 1. Specifically, a center bias was in heading perception, and the previous uniform heading distribution did not affect heading perception from optic flow.

## Experiment 3. Heading Distribution Affects Heading Perception from Optic Flow

Experiments 1 and 2 found that the uniform distribution did not affect heading perception from optic flow, and the central tendency was not in heading perception from optic flow. As mentioned in the introduction, previous studies have demonstrated that the central tendency in other feature perceptions can be characterized by a Bayesian ideal observer model in which the prior feature distributions work as the priors (Ashourian & Loewenstein, 2011; Jazayeri & Shadlen, 2010; Karaminis et al., 2016; Olkkonen & Allred, 2014). Theoretically, this model could also explain the findings of Experiments 1 and 2 (please see the equations in the Introduction for details). Hence, we could not directly conclude that heading distribution did not affect heading perception from optic flow.

**Figure 6.**
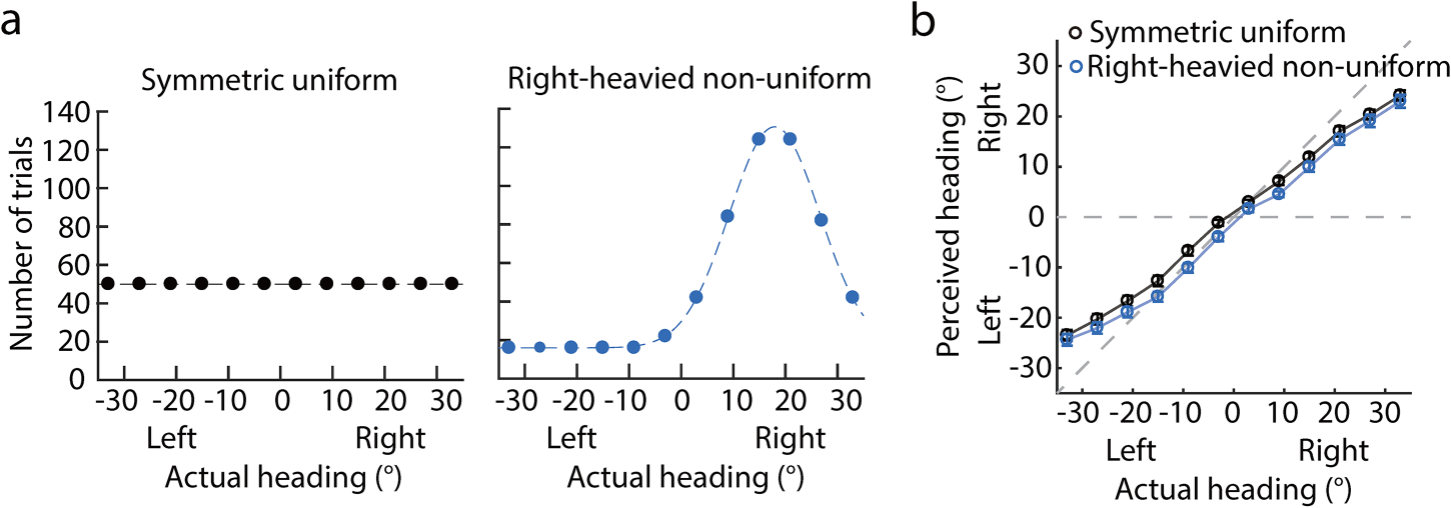
(a) Number of trials against the actual heading. Left and right panels show the symmetric uniform and right-heavied nonuniform distributions. (b) Results of Experiment 3. The perceived heading (*y*-axis) was against the actual headings (*x*-axis). The left and right on the *x*-axis (*y*-axis) mean the actual heading (perceived heading) was left or right in the straight-ahead direction (0°). Black and blue circles represent the results of symmetric uniform and right-heavied nonuniform distributions. Each circle represents the mean perceived heading averaged across 20 participants; error bars represent the standard error across 20 participants.

To reexamine whether heading distribution affected heading perception from optic flow, we showed 20 new participants (10 females, 10 males, age 18–23 years) with a symmetric uniform distribution and a right-heavied nonuniform distribution (Figure 6a). In the right-heavied nonuniform distribution, the headings were randomly selected from ±33°, ±27°, ±21°, ±15°, ±9°, or ±3°, while the trial numbers for headings −33°, −27°, −21°, −15°, −9°, −3°, 3°, 6°, 9°, 15°, 21°, 27°, and 33°are 16, 16, 16, 16, 16, 22, 42, 84, 124, 124, 82, and 42, leading to more heading appeared at the right side of the ego-centric direction with a 12.05° distribution mean, right to the display center. With the nonuniform distribution, the $p_{\mathrm{dis}}(X|\theta_{\mathrm{dis}})$ varied among headings, so $p_{\mathrm{dis}}(X|\theta_{\mathrm{dis}})$ could not be eliminated from equation (2). As a result, participants’ PHs were affected by both the ego-centric direction and the prior heading distribution. The stimulus parameters and experimental design were similar to that of Experiment 1. The experiment lasted for about 50 min.

### Results and Discussion

Like Experiments 1 and 2, we also first fitted the PH as a linear function of the AH to examine whether center bias was in heading perception. One sample *t*-test showed that the slopes of the symmetric uniform and right-heavied nonuniform distributions were all significantly smaller than 1 (mean ± SE: 0.75 ± 0.035, *t*[19] = −7.17, *p* < .001, Cohen's *d* = 1.60; 0.75 ± 0.035, *t*[19] = −6.98, *p* < .001, Cohen's *d* = 1.56), indicating a center bias in heading perception, consistent with Experiments 1 and 2.

Figure 6b plots the PHs against the AHs in the symmetric uniform (black circles) and right-heavied nonuniform (blue circles) distributions. It shows that compared with the symmetric uniform distribution, the overall PHs of the right-heavied nonuniform distribution were overall biased toward the left side where headings rarely appeared. A 2 distributions (symmetric uniform vs. right-heavied nonuniform) × 12 headings repeated measures ANOVA showed that the main effects of distributions were significant (*F*[1,19] = 9.64, *p* = .0058, *η*^2^ = 0.337). Specifically, compared with the PH of the 0°-mean uniform distribution (mean ± SE: 0.20 ± 0.30, 95% CI: [−0.43, 0.84]), the PH of 12°-mean right-heavied distribution (mean ± SE: −1.80 ± 0.51, 95% CI: [−2.86, −0.73]) was more left-biased. The interaction between distributions and headings was not significant (Greenhouse–Geisser corrected: *F*[1.86,35.29] = 0.81, *p* = .63, *η*^2^ = 0.041). The results showed that compared with symmetric uniform distribution, the PHs in the right-heavied nonuniform distribution were overall biased toward the left side, where headings rarely appeared. This finding suggested that the central tendency was not in heading perception, but observers learned the previous heading distribution and used it to adjust their heading perceptions.

The heading bias towards the side where headings rarely appeared indicated that the PH was overall biased away from the distribution center, showing a repulsive effect of heading distribution. Previous studies suggested that the central tendency in other feature perceptions was consistent with a Bayesian inference account in which the previous heading served as a prior (Ashourian & Loewenstein, 2011; Jazayeri & Shadlen, 2010; Karaminis et al., 2016; Olkkonen & Allred, 2014). However, the Bayesian inference account was only applicable to the attractive bias but not to the repulsive bias, which was further discussed in the general discussion part.

Sun et al. (2020) found that the perception of the current heading was biased away from the headings of previous trials after removing center bias, showing a repulsive serial dependence effect (also see Sun & Li, 2019). The headings in their experiments were selected from a geometric progression (i.e., ±32°, ±16°, ±8°, ±4°, ±2°, and 0°), and each heading was repeated for the same trials. This setup leads to a nonuniform heading distribution, similar to our right-heavied distribution in the current study. Hence, we proposed that the repulsive effects of nonuniform distribution on heading perception might be due to the repulsive serial dependence. When more previous headings were from the right side, the PH would be repulsed to the left side. If this was one of the reasons, then a repulsive serial dependence would be observed in the right-heavied nonuniform distribution but not in the symmetric uniform distribution.

**Figure 7.**
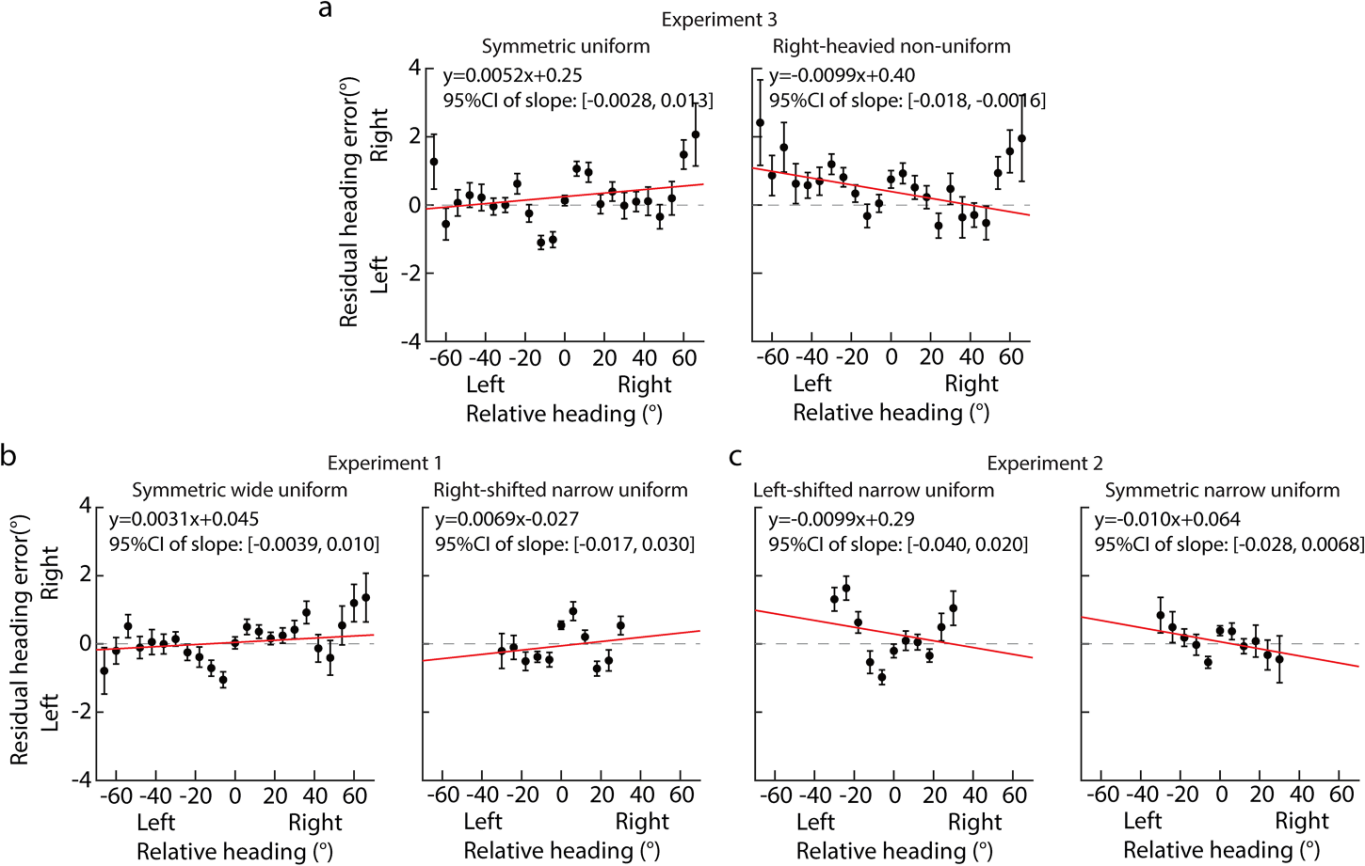
Serial dependence results. The residual heading error (*y*-axis) was against the relative heading (*x*-axis). The dot indicates the mean residual heading error across the 20 participants; the error bar indicates the standard error across the 20 participants. Red lines are the best linear fitting between residual heading error and relative heading. (a) Results of symmetric uniform (left panel) and right-heavied nonuniform (right panel) distributions in Experiment 3. (b) Results of symmetric wide (left panel) and right-shifted narrow (right panel) uniform distributions in Experiment 1. (c) Results of left-shifted (left panel) and symmetric narrow distributions in Experiment 2.

To test the above hypothesis, we analyzed the serial dependence with the method of Sun et al. (2020). Specifically, for each participant, we first fitted the PH as a linear function of the AH, given as $\;\hat{\mathrm{PH}}=s\;\mathrm{timesAH}+\mathrm{error}$. $\hat{\mathrm{PH}}$ was the predicted PH. The difference between the actual and predicted PH was proposed to be from the serial dependence, named as the residual heading error (RHE). Then, on group level, we fitted the RHE as a linear function of the relative heading (RH) that was the difference in AH between the previous first trial and the current trial, given as $\mathrm{RHE}=s^{\prime }\;\times \mathrm{RH}+\mathrm{error}$. If $s^{\prime }$ was negative, then a repulsive serial dependence was in heading perception (please see Sun et al. 2020 for more details). Figure 7a shows the serial dependence results of current experiment. It clearly showed that a repulsive serial dependence was in the right-heavied nonuniform distribution (linear fitting: $s^{\prime }$ = −0.0099, 95% CI: [−0.018, −0.0016]) rather than in the symmetric uniform distribution (linear fitting: $s^{\prime }$ = 0.0052, 95% CI: [−0.0028, 0.013] in Experiment 3). Serial dependence effects were also not in Experiments 1 and 2 in which the headings were uniformly distributed (Figure 7b and 7c).

With the results of the serial dependence analysis in Experiment 3, we calculated the difference in heading bias between the uniform and nonuniform distributions caused by the serial dependence effect that was about −0.031°, accounting for about 1.57% of the total heading bias difference between the two heading distributions (i.e., −1.98°). Therefore, the repulsive serial dependence only explained a tiny fraction of the repulsive effect of heading distribution.

## General Discussion

The current study conducted three experiments to examine whether and how heading distributions affected heading perception from optic flow. Specifically, we investigated whether a central tendency was in heading perception from optic flow. Experiments 1 and 2 showed that when heading distributions were uniform, headings were systematically biased toward the ego-centric direction, showing a center bias (Sun et al., 2020; Xing & Saunders, 2016). Additionally, the PHs were not significantly different between different uniform distributions, suggesting that uniform distribution did not affect heading perception. However, aside from reproducing center bias, Experiment 3 showed that compared with the PHs in the uniform distribution condition, the PHs were overall biased toward the side where headings rarely appeared in a nonuniform distribution. Together, the results suggested that heading perception from optic flow was center-biased, but no central tendency was in heading perception, irrespective of whether heading distributions were uniform or nonuniform. Even so, the current study showed that the heading distribution affected heading perception from optic flow.

Previous studies have revealed that central tendency occurs across a variety of physical features, for example, object size (Hollingworth, 1910), line length (Ashourian & Loewenstein, 2011; Duffy et al., 2010; Huttenlocher et al., 2000), absolute size (Huttenlocher et al., 2000), time interval estimation (Jamieson, 1977; Jazayeri & Shadlen, 2010; Ryan, 2011), and color (Olkkonen & Allred, 2014; Olkkonen et al., 2014). However, the current study found that the central tendency was not in heading perception from optic flow, indicating that the central tendency was not in all physical feature perceptions.

The absence of a central tendency in heading perception from optic flow could be attributed to the complex optic flow stimulus. Previous central tendency studies generally used basic physical features, such as color, size, length, and even time interval. In contrast, optic flow is the integration of multiple different motion directions. Previous studies reported that a central tendency was found in facial expressions (Horstmann, 2002), suggesting that the central tendency could exist in complex feature perception. Hence, the complex feature explanation might be untenable.

Another reason could be that optic flow is dynamic. Color, size, length, and even facial expressions are all static stimuli. Hence, we proposed that the central tendency might not be in dynamic feature perceptions. So far, no research has been conducted to examine whether the central tendency is in motion perception, which remains to be tested.

Although we did not observe a central tendency in heading perception from optic flow, the results showed that when the heading distribution was nonuniform (right-heavied), PHs would be biased toward the side where headings rarely appeared. In other words, the PHs have shifted away from the heavily distributed side, indicating that the nonuniform heading distribution would repulse the heading perception. Sun et al. (2020) used a nonuniform heading distribution and found that the perception of the current heading was biased away from the previous first trial, showing a repulsive serial dependence effect (also see Sun & Li, 2019). Using their methods, we found that a repulsive serial dependence was in the right-heavied nonuniform distribution rather than the uniform distribution, indicating that the heading distribution modulated serial dependence in heading perception from optic flow (future studies could further examine this finding). Importantly, the repulsive serial dependence only accounted for a tiny fraction of the repulsive effect of heading distribution, suggesting that other reasons could lead to the heading distribution effect.

One might be the phenomenon of the “prevalence-induced concept change (PICC).” PICC means that, compared with recent experience, when the probability of occurrence of one concept was reduced (or increased), observers tended to mistakenly infer that the probability of occurrence was increased (or decreased), resulting in observers reporting more features from the rare side (Levari et al., 2018). Levari et al. found that the PICC occurred across a variety of feature perceptions, such as color, facial valiance (threatening vs. nonthreatening), and Institutional Review Board ethical judgment. In the current study, when more headings recently experienced were from the right side of the ego-centric direction, participants might mistakenly increase the probability of the left-side headings and report the headings as more left-biased, consistent with the PICC. Moreover, all headings occurred with the same probability in the uniform distribution in a certain range, the PHs, therefore, would not be biased toward one side.

Previous central tendency studies with other features showed that a Bayesian inference model could predict the central tendency in which the previous feature distribution served as the prior (Ashourian & Loewenstein, 2011; Jazayeri & Shadlen, 2010; Karaminis et al., 2016; Olkkonen & Allred, 2014). This model can theoretically characterize participants’ PHs in previous normal distributions (please see the derivation of equations [1] and [2] in the Introduction). However, because the Bayesian inference model can only predict the performance biased toward the previous distribution mean, it cannot characterize the trend that the PHs were biased toward the side where headings rarely appeared in nonuniform distribution. Recently, several researchers proposed an efficient coding model that successfully predicts the “anti-Bayesian” performance of various physical feature perceptions (see Wei & Stcoker, 2017). The efficient coding model assumes that the neural system allocates its neural system resources efficiently to maximize the mutual information between internal space and the outside physical world. That is, based on the prior frequency information of the stimuli, the neural system encodes the stimulus appearing most frequently (high probability prior region) in the real world with high certainty while encoding the stimulus appearing less frequently (low probability prior region) with low certainty. When a stimulus is presented, the neural system has high certainty to judge whether a stimulus belongs to the high probability prior region. As a result, any stimulus that does not belong to the high probability prior region will be inferred to be from the low probability prior region. For a nonuniform prior distribution with low probability on tails and high probability at the center (like the right-heavied distribution in Experiment 3), the perceived features would be biased away from the prior distribution mean, showing a symmetric peripheral bias. However, the PHs were biased toward the left side where the heading rarely appeared in the right-heavied distribution condition, not a symmetric peripheral bias. Hence, the efficient coding model cannot characterize the repulsive effect of heading distribution on heading perception from optic flow. At the end of this paragraph, we have to admit that we fail to figure out an effective computational model to predict our findings, so this is an open question for future studies.

Xing and Saunders (2016) examined whether the center bias in heading perception was due to perceptual bias or response bias by presenting participants with one uniform distribution and one M-shape distribution. In the M-shape distribution, the trial number linearly increased with the heading. If a response bias was in the heading perception, then the PHs would be biased toward the peripheral side where more headings appeared, leading to a decrease in center bias. They found that center biases were in the heading perception but were not significantly different between the two distributions. Therefore, they concluded that the heading bias was perceptual rather than response. To some extent, the current study supported their conclusions that heading bias was not a response bias. Specifically, if the heading bias was due to response bias, then the PHs would be more right-biased in the right-shifted narrow uniform distribution and right-heavied nonuniform distribution. However, the expected right-side bias was not in the distributions, suggesting that heading perception was not response bias.

Furthermore, our current study did not support Xing and Saunders’ idea that the heading bias was perceptual. Because we found that observers biased their PH away from the side where more headings appeared in the right-heavied nonuniform distribution, it suggests that participants were aware of and learned the heading distributions. They adjusted their heading perception using the learned heading distribution, indicating that postperceptual stages (e.g., attention and working memory) were involved in heading perception from optic flow. Therefore, the heading bias was not purely perceptual.

Previous studies have demonstrated that PHs are the optimal combination of the ego-centric direction and the current heading (D’Avossa & Kersten, 1996; Li et al., 2002; Warren & Hannon, 1988; Warren & Kurtz, 1992; Warren & Saunders, 1995). The neurons in cortical areas VIP and MST encode the headings simulated by optic flow with the ego-centric direction (Chen et al., 2011, 2018). It follows from the above that combining the ego-centric direction and the current heading does not take the cognitive resources (e.g., attention and working memory). However, our current study revealed that the heading perception from optic flow involves postperceptual stages, which is inspirational for future studies. The effect of experience on heading perception, in particular, implies that the heading perception from optic flow involves a top-down processing mechanism. High-level cognitive abilities (e.g., attention, working memory) may be involved in the heading perception. Recent neurophysiological and electrophysiological studies mainly examine the roles of low-level cortical areas in heading perception but ignore the roles of high-level cortical areas, such as prefrontal cortex (PFC) that is responsible for working memory (Collette & Linden, 2002; Müller & Knight, 2006) and the frontal and parietal cortex that are responsible for attention (Kastner & Ungerleider, 2000). Starting with the current study, we can develop a series of psychophysical and neuroscience experiments to examine the effects of high-level cognitive functions (e.g., working memory, attention) in heading perception.

To summarize, the current study revealed that while the central tendency was not in heading perception from optic flow, observers did summarize the previously seen headings from optic flow and used it to judge their headings, suggesting that postperceptual stages (e.g., attention and working memory) could be involved in heading perception. In other words, heading perception from optic flow was not purely perceptual.
